# Creative tensions: mutual responsiveness adapted to private sector research and development

**DOI:** 10.1186/s40504-017-0058-6

**Published:** 2017-09-07

**Authors:** Matti Sonck, Lotte Asveld, Laurens Landeweerd, Patricia Osseweijer

**Affiliations:** 1 Faculty of Applied Sciences, Delft University of Technology, Van der Maasweg 9, 2629HZ, Delft, the Netherlands; 2 Faculty of Science, Institute for Science, Innovation and Society, Radbourg University Nijmegen, Heyendaalseweg 135, 6525AJ, Nijmegen, the Netherlands

**Keywords:** RRI, Responsible research and innovation, Responsible innovation, Responsiveness, Research and development, Innovation ethics, Innovation management, Stakeholder engagement

## Abstract

The concept of mutual responsiveness is currently based on little empirical data in the literature of Responsible Research and Innovation (RRI). This paper explores RRI’s idea of mutual responsiveness in the light of recent RRI case studies on private sector research and development (R&D). In RRI, responsible innovation is understood as a joint endeavour of innovators and societal stakeholders, who become mutually responsive to each other in defining the ‘right impacts’ of the innovation in society, and in steering the innovation towards realising those impacts. Yet, the case studies identified several reasons for why the idea of mutual responsiveness does not always appear feasible or desirable in actual R&D situations. Inspired by the discrepancies between theory and practice, we suggest three further elaborations for the concept of responsiveness in RRI. Process-responsiveness is suggested for identifying situations that require stakeholder involvement specifically during R&D. Product-responsiveness is suggested for mobilising the potential of innovation products to be adaptable according to diverse stakeholder needs. *Pre*sponsiveness is suggested as responsiveness towards stakeholders that are not (yet) reachable at a given time of R&D. Our aim is to contribute to a more tangible understanding of responsiveness in RRI, and suggest directions for further analysis in upcoming RRI case studies.

## Introduction

There are calls on companies to respond to the needs of societies within which they operate, beyond securing short-term profitability and complying with regulations. In Europe, this call has recently been voiced in the field of *Responsible Research and Innovation* (RRI), a research policy approach that has been coined in the European Commission’s policy context as the most recent framework to address societal dimensions of science and technology. RRI builds on the one hand on its earlier research policy counterparts, such as ELSA (ethical, legal and social aspects). On the other hand, RRI is developed further through several emerging research approaches that can be captured under the heading of ‘responsible innovation’. From these premises, RRI posits that Research and Development (R&D) processes should anticipate and reflect societal aspects of the innovation, but also that innovators are expected to be *responsive* to these considerations by adjusting the shape (e.g. design) and direction of the innovation (Owen et al., 2013; Stilgoe, Owen, & Macnaghten, 2013). Furthermore, stakeholder involvement is a substantial element in all RRI approaches (Koops, 2015). It is emphasised that R&D should be an inclusive process, involving interaction between innovators and societal stakeholders, who become *mutually responsive ‘*to each other with a view to the (ethical) acceptability, sustainability and societal desirability of the innovation process and its marketable products’ (von Schomberg, 2013).

Increasing attention within the RRI community is now turning towards private sector R&D. Given that RRI challenges both innovators and stakeholders to be active contributors to the responsibility of innovation processes and its outcomes, the question arises, how their interaction can best be organised to enable *mutual responsiveness*. As Stirling already claimed in 2008, stakeholder involvement is about *opening-up* the innovation to ‘participatory deliberation’ about its goals and purposes in society. RRI posits that in the course of this process, the innovators and stakeholders would become *mutually responsive,* implying that they reach some form of a joint understanding about how the innovation is shaped, and eventually applied. Deliberation can then be *closed-down* and decisions made in order to move on with the innovation (Stirling, 2008).

So far, the understanding of mutual responsiveness in RRI has been criticised for being highly naive: as unconcerned about private sector characteristics. In particular, it is assumed that innovators and stakeholders engage continuously in a transparent process, and also end up sharing responsibility. In reality, corporate innovation is characterised by high investment and risk imbalances, as well as power and information asymmetries. (Blok & Lemmens, 2015) What are the chances of *opening-up* the innovation to participatory deliberation in face of such asymmetries? Further, understanding of mutual responsiveness appears highly demanding in its optimism about resolving the discrepancies between stakeholder needs and perspectives. To become mutually responsive requires learning, interdependence, trust to take place among actors with very different needs and interests. (Nielsen, 2016) How can we *close-down* the deliberation in face of these differences? These shortcomings partly indicate that RRI’s conceptualisation is still open-ended, with little detailed description of what mutual responsiveness could imply in practical innovation contexts (with exceptions like Blok (2014) and Haen et al. (2015)). Furthermore, RRI and its predecessors have been mainly developed in policy and academic contexts (Blok & Lemmens, 2015; Scholten & van der Duin, 2015), and the literature on stakeholder involvement largely centres around public policies and science governance (cf. Delgado, 2010; Ganzevles & van Est, 2012). These contexts may partly capture different problematics and opportunities than the company environment

Recently, Blok et al. (2015), Noorman et al. (2017) and Blok et al. (2017) have each explored how private sector R&D complies with RRI’s ideas. While these case studies conclude that the case companies fell short of the idea of mutual responsiveness via continuous multi-stakeholder collaboration, they also bring out ‘reasonable reasons’ for why such collaboration is not always possible – or desirable. What is more, the studies portray alternative management practices to interrogate stakeholders’ perspectives, and respond to those in the course of R&D.

This paper draws inspiration from the discrepancies between RRI’s idea of mutual responsiveness, and how stakeholders were actually involved in private sector R&D in these recent case studies. Our main question is: How could responsiveness be operationalised in R&D, given the limitations of mutual responsiveness identified in practical innovation environments? By paralleling RRI-related theory and practice, we will suggest three further elaborations for the concept of responsiveness as an answer to our question. **Process-responsiveness** is suggested for identifying situations, which particularly require opening-up of the innovation at R&D level. **Product-responsiveness** is suggested for mobilising the potential of R&D’s products to be adaptable to diverse stakeholder needs. ***Pre*** **sponsiveness** is suggested as responsiveness towards stakeholders that are not (yet) reachable at the time of R&D. The aim in presenting these elaborations is to contribute to a more tangible concept of responsiveness in RRI, while also suggesting directions for analysis in upcoming case studies. Comparing

The article will unfold as follows. Section 2 introduces theoretical background and the case studies. In section 3, we will discuss the tensions between theory and practice in a more detail, and as an outcome suggest the elaborations for the concept of responsiveness. In conclusions (Section 4), we briefly reflect on generalizability and limitations of the outcomes.

## RRI and responsiveness

The term ‘responsiveness’ embodies many core elements of RRI’s conception for responsible innovations. As the *action element* of RRI, responsiveness mobilises the societal input into explicit actions in innovations, so that the innovation becomes better aligned with societal needs (Flipse et al., 2015; Owen et al., 2013). Further, responsiveness as *forward-looking responsibility* signifies a ‘receptive attitude’ of reacting and responding to new knowledge as it emerges, while acknowledging the uncertainty and limited control that are inherent to innovations (Pellizzoni, 2004; Stilgoe et al., 2013). From this standpoint, societal challenges appear as positive triggers for socio-economic improvements, which according to RRI are attainable through innovations, provided that there are (continuous) efforts to discuss and define societal ‘right impacts’ and ‘right processes’ for their implementation (Zwart et al., 2014). Furthermore, to be responsive also embodies a *relationship* between innovators and societal stakeholders. *Mutual responsiveness* highlights reciprocity and proactivity in this relationship, in that the actors are expected to jointly shape and direct the innovation towards realising the ‘right impacts’. This definition excludes, for instance, unidirectional ‘pushing’ of information to public about latest technical advances, or ‘pulling out’ valuable knowledge or confidence about acceptability from the public (Lee & Petts, 2013; Stirling, 2008).

To become mutually responsive, innovators and different stakeholders are first expected to recognise differing perspectives on the innovation, and then to become attentive to others’ perspectives – and critical of their own. This would lead to a form of a joint understanding, such as consensus, agreement on courses of action (Asveld & Stemerding, 2017), alignment of expectations, acceptance of conflict (agreeing to disagree) (Blok et al., 2015), or re-constructing of the self (cf. Blok, 2014, for dialogical responsiveness). Hence, mutual responsiveness demands reflexivity and learning between actors with different interests, trust and interdependence, as well as commitment to jointly find long-term solutions to societal challenges (Flipse et al., 2014; Nielsen, 2016). From innovators, mutual responsiveness asks readiness to provisionally acknowledge the legitimacy of raised concerns (Haen et al., 2015). From stakeholders, it requires a constructive input in terms of defining what is societally desirable (von Schomberg, 2013), and hence willingness to think and speak about concerns (Haen et al., 2015). Not the least demanding, mutual responsiveness is described as resulting from continuous and transparent exchange of information (e.g. via stakeholder dialogue), and is assumed to lead to sharing responsibility among the actors (von Schomberg 2013; Blok et al. 2015).

### Mutual responsiveness: *why*, *how*, *with whom*

Several challenges regarding RRI’s ideas of multi-stakeholder activities have been identified. With regard to backward-looking responsibilities (Pellizzoni, 2004), there are for instance concerns whether blurring of role differentiation would lead to unclear distribution of accountability (Landeweerd, 2017; Zwart et al., 2014). With our focus on responsiveness (i.e. forward-looking responsibility), we assume in this paper that accountability remains with the innovator. We also assume this, since companies (investing in new innovations) and their stakeholders seem to agree that the investor alone is responsible, when it comes to making investment decisions (Blok et al., 2015). Focusing on responsiveness, we will thus elaborate challenges faced by ideas of *mutually responsive* relations among innovators and stakeholders. To mobilise further theories for discussing the challenges identified in the case studies, we pose three guiding questions about mutual responsiveness.

Our first question is: *Why should the private sector R&D and stakeholders become mutually responsive?* In Section 3.1, we will reflect on RRI’s idea of frequent stakeholder involvement against situations, where companies (allegedly) were already responsive to societal needs without a need for such involvement. These situations bring up two distinctive, but not mutually exclusive, approaches in RRI on how to operationalise responsiveness in innovations (Blok et al., 2017). In the more *normative approach*, innovation can be responsive by applying normative ‘anchor points’ (von Schomberg, 2013) as its goals, such as sustainability or public health. The normative approach builds on substantive rationale, in the sense that the reason for involving stakeholders is to obtain better results, such as improved public health (Delgado et al., 2011; Fiorino, 1989; Stirling, 2008). Correspondingly, the processes are less fixed and thus amenable to adjustments according to their relevance for the outcome. On the other hand, *procedural approach* posits that responsible innovation *is* a deliberative and inclusive process (Blok et al., 2017). The rationale is in procedural norms: stakeholder involvement is ‘the right thing to do’ for the sake of the process (e.g. following an ideal of democracy) (Delgado et al., 2011; Fiorino, 1989; Stirling, 2008). Thus, outcomes are less fixed and more amenable to influence by the public demand (Blok et al. 2017). An application of *procedural approach* is also the framework by Owen et al. (2013) whereby responsible innovation is a process of inclusive anticipation and reflection, resulting in a response steering the innovation.

Second, if the innovation is to be opened-up: *How can the private sector R&D and stakeholders become mutually responsive?* Section 3.2 will bring up several limitations that stood out in the case companies’ efforts for involving stakeholders during R&D. Further, case studies display an array of management practices for involving stakeholders – given these limitations. We highlight the need to consider these practices in the context of their purpose. For this, we evoke Stirling’s (2008) distinction between *appraisal* (i.e. informing decision making) and *commitment* (forming tangible decisions on particular innovation trajectories). Appraisal and commitment can involve both *opening-up* as well as *closing-down* the innovation. *Opening-up appraisal* can provide ‘plural advice’ for innovators, as it welcomes diverging societal discourses and framings in the discussion, and weighs alternative courses of action. In contrast, *closing-down appraisal* is prone to support decision makers’ ‘incumbent interests’ and instrumental behaviour: discussion already excludes alternative framings and courses of action in advance. In the time of commitment, some degree of closing down is necessary and desirable in order to move on, but Stirling also remarks that this *closing-down commitment* tends to be ‘unduly privileged’. He suggests that consideration should also be given to *open-ended commitments*, as they leave space for diversity, and promote context sensitivity, avoidance of lock-ins, and social learning.

The third question is: *With whom should the private sector R&D become mutually responsive*? Section 3.3 will discuss situations, in which opening-up the innovation for stakeholder engagement was perceived as non-informative during early steps of R&D, indicating also uncertainty about who should count as a stakeholder. Here, we return to the definition of responsiveness as future-oriented responsibility, which obliges a ‘receptive attitude towards needs and desires of others, before deciding what to do’ (Pellizzoni, 2004). Yet, how can there be mutual responsiveness among the innovators and those actors, who are potentially affected by the innovation but are not available at the context of R&D? We approach this question in view of the Collingridge dilemma (Collingridge, 1980) that has been widely discussed in RRI-related literature (e.g. Blok & Lemmens, 2015; Flipse et al., 2013; Owen et al., 2012). That is: In its early steps an innovation would be better amenable for modifications based on stakeholder input, but there is not enough knowledge for grasping the impacts of the innovation on society. Conversely, by the time the concept is explicit enough to allow diverse societal reflections, it is already locked-in to certain trajectories so that steering the innovation is difficult, costly and time consuming.

### Case studies

We will discuss these guiding questions principally based on three case studies from RRI literature: one from the ICT sector (Noorman et al., 2017) and two from the food sector (Blok et al., 2015; Blok et al., 2017). These studies were chosen as they are ‘exploratory’: They examine decision-making in private sector R&D from RRI perspectives, based on actual data from the companies (interviews, surveys, observation). Furthermore, the studied companies are aiming to address societal challenges with their innovations, thus having ‘societal aspirations’ (Noorman et al., 2017) and ‘disposition to innovate more responsibly’ (Blok et al., 2015). Furthermore, their stakeholders include non-commercial actors, in addition to commercial partners.

Noorman et al. (2017) introduce a start-up with a pseudonym **Datashare**, developing an online digital platform that would allow residents, government organisations, and service providers to exchange information about energy consumption. Datashare aims to develop the platform for ‘privacy-friendly data sharing’, enabling both the resident-users to control their own data, and the business partners to access the resident data. With this aim, Datashare needs to balance between conflicting interests and values (privacy and access) of their key stakeholders. To address this conflict, Noorman and colleagues proposed a stakeholder workshop, inviting residents, business partners, and privacy-oriented civil society organisations (CSOs), to jointly reflect upon implicit values, biases and interests regarding the platform. This proposal was dismissed by Datashare, which led the authors to explore ‘reasonable reasons’ restricting stakeholder involvement. Further, it led the authors to explore how Datashare attempted to be responsive to stakeholder needs and values within these restrictions, through ‘tinkering and improvisation’.

Blok et al. (2015) studied several **Dutch food companies** and their non-commercial stakeholders, in order to find out to what extent companies with a disposition to innovate more responsibly are moving towards the idea of mutual responsiveness. For this, the authors examined to what extent companies engage stakeholders at different steps of the innovation process. They conclude, that the companies fall short of the ideal of mutual responsiveness as a transparent and interactive relation leading to sharing responsibility. Stakeholder engagement was not continuous, as it mostly took place at strategic level and early R&D phase (idea generation), and sometimes as an ‘extra-check’ in the late (commercialisation) phase. In the middle (developmental) phase, stakeholders were rarely involved and only under strict intellectual property conditions. The authors then identified several critical issues restricting transparency, interaction, responsiveness and co-responsibility in private sector R&D settings. Moreover, several management practices to deal with these critical issues were identified.

Blok et al. (2017) studied food companies that participate in a front-of-package **(FoP)** logo for healthier food products. The authors explored, to what extent the companies contributing to global health challenges consider social-ethical factors in their R&D. By applying the stage-gate model (Cooper, 1990), and Jones’s (1991) theory of ethical decision making, the authors conclude that ethical decision making did not occur at any step of the R&D process. Further, stakeholders were not involved in the decision making process during R&D. However, the authors suggest that ethical decisions, such as trade-offs between health benefits and techno-economic factors, had possibly been made at a higher strategic level, where stakeholders like health organisations could also have been involved. These strategy-level decisions then set boundary conditions for R&D, within which R&D then focuses on techno-economic factors (e.g. quality, costs).

From here on, these cases will be referred to as **Datashare case**, **Dutch food case**, and **FoP case**, respectively. Due to the small number of cases, we also refer to a number of *background case studies* in the RRI literature, which are not ‘exploratory’ in every aspect of our definition, but can further elucidate the findings. Asveld & Stemerding (2017) describe a case in which companies developing a bio-based cleaning product were targeted by a critical campaign by environmental CSOs. The authors illustrate how mutual learning among stakeholders could have been organised during the R&D process, in order to unveil differing notions on what is ‘sustainable’. Balkema & Pols (2015) investigate negative socio-economic and environmental impacts of biofuel crop cultivation in Tanzania, affecting the hardest the most vulnerable stakeholders, the small farmers. By means of an ethical framework the authors identify responsibilities of each stakeholder, concluding that such identification during stakeholder engagement would have been precondition for a sustainable biofuel innovation. Dignum et al. (2016) studied stakeholder argumentation for and against shale gas exploitation in the Netherlands, based on which they examine applicability of Value-Sensitive Design (VSD) in the design of stakeholder participation processes. Haen et al. (2015) organised public engagement exercises around novel food products, while developing a tool to unveil and address ethical, cultural and political concerns that often appear to be overlooked in food innovations. Scholten and van der Duin (2015) studied the extent to which spin-off companies from academia are applying elements of responsible innovation. In a survey of a sample of start-ups in the Netherlands, the authors’ findings included that ‘social responsiveness’ (inclusion of the social aspects of what the firm produces and develops in the innovation) increases the companies’ capacity to absorb external knowledge, and to apply that knowledge in their innovations. Finally, van den Hoven (2013) discusses public debates around smart electricity meters and electronic healthcare records, and reflects on the potential of VSD to make conflicting values (e.g. privacy, resource efficiency, access) explicit and accommodated in the product design.

## Implementing mutual responsiveness in the private sector

**Fig. 1:**
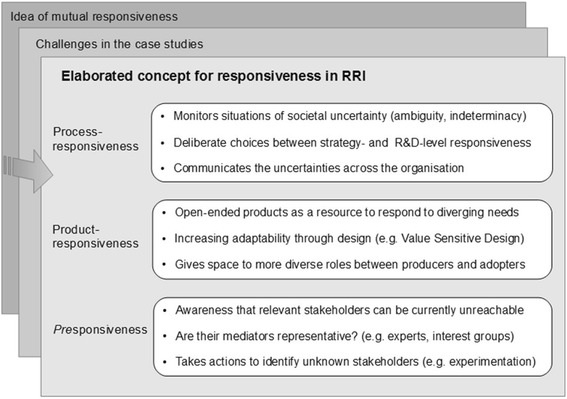
Three elaborations for the concept of responsiveness in RRI

This section suggests process-responsiveness, product-responsiveness, and *pre*sponsiveness as further elaborations for the concept of responsiveness (See Fig. 1). Before each elaboration, we first describe **limitations** that stood out in case studies as challenging RRI’s idea of mutual responsiveness. Namely, the studied companies perceived several ‘critical issues’ (Blok et al., 2015) and ‘reasonable reasons’ (Noorman et al., 2017) limiting stakeholder collaboration. After each elaboration, we present **discussion** that led to our suggestions. The discussion reflects RRI theories with ‘management practices’ (Blok et al., 2015) that the companies applied for dealing with the challenges in their stakeholder collaboration.

### Why become mutually responsive: Process-responsiveness

#### Limitation: No perceived need for mutually responsive R&D

The case companies did not always perceive a need to consider societal aspects of their innovation at R&D level, nor involve stakeholders for this. Instead, they pursued their societal aspirations by other means. Both the FoP companies (Blok et al., 2017) and Dutch food companies (Blok et al., 2015) applied healthy food criteria agreed upon by their stakeholders, as mandatory boundary conditions for the operational R&D. Within these boundaries, the R&D then focused merely on techno-economic issues. The decision to adopt these criteria had been made at the corporate strategy level – possibly involving also stakeholders like health organisations. In addition, Dutch food companies organised stakeholder rounds during early R&D, but expressed that frequent stakeholder involvement was often not necessary after the early steps, as ‘science does not change every week’ and stakeholder opinions hardly change that suddenly (Blok et al., 2015).

#### Suggestion: Process-responsiveness

These findings are in line with recent conclusions that Corporate Responsibility approaches often receive little consideration at the R&D level. While companies have adopted strategies to address societal and environmental impacts of their operations, such as Corporate Social Responsibility (CSR) (cf. Iatridis & Schroeder, 2016; Pellé & Reber, 2015), social and ethical aspects are still not usually included in the ‘throughput’ (Blok & Lemmens, 2015), or ‘midstream’ (Flipse 2012), of innovation processes. This bears a risk of discrepancies forming between strategic and operational levels (Blok et al., 2017). Furthermore, there are retrospective studies on unsuccessful projects suggesting that *opening-up* the innovation to stakeholder perspectives during the R&D process could have enhanced both the acceptability and commercial success of the project (e.g. Asveld & Stemerding, 2017.; Dignum et al., 2016).

Against this background, we suggest the **process-responsive** approach as a step to further operationalise responsiveness in innovations. Process-responsive innovation:

- Makes deliberate choices between adopting a more normative (strategy-level) or a more procedural (R&D-level) approach to responsible innovation.
- Considers the extent of uncertainty in making these choices. When the normative approach is followed, remains alert to uncertainties that call for opening-up the innovation at the R&D level to wider reflections on its goals and purposes. Such situations include, among others, application of emerging technologies (high ambiguity) and radical innovations (indeterminate uncertainty).
- Encourages communication between R&D and the strategy level about the uncertainties, for example via organisational culture and structures that support such interaction.

Process-responsiveness also makes RRI more explicit about what is expected of company innovators, if they are to become mutually responsive with societal actors with a view to the societal aspects of the innovation (von Schomberg, 2013). Simultaneously, it further elaborates responsiveness as the *action element* of RRI, by suggesting the deliberate choice between normative and procedural approach as one form of such action.

#### Reflecting RRI and practice

As Blok et al. (2017) note, FoP companies’ practices run contrary to some of RRI theory expectations. One the one hand the companies were responsive to the societal need for healthier food, by following criteria (e.g. salt and calorie levels) that are in line with normative societal goals (public health). In this normative sense, they were attentive to the ‘right impacts’ of the innovation regarding the impact of their products (Blok et al., 2017; Owen et al., 2012; von Schomberg, 2013). Yet, their approach was inconsistent with the procedural approach: R&D did not anticipate societal impacts or reflect purposes of the innovation, to any extent identifiable in their decision making (Blok et al., 2017). Just as little was there any inclusive opening-up of the innovation during R&D to the perspectives of societal actors (Owen et al., 2012; Stirling, 2008), and hence no mutual responsiveness at the R&D level.

However, Blok et al. (2017) suggest that companies had weighed trade-offs between ethical and techno-economic aspects, such as between salt level and shelf-life, at the corporate strategy level. Furthermore, stakeholders like health organisations may have had an influence on the companies’ innovation agendas via strategy-level dialogue, although this was out of the scope of the FoP study. Thus, there appears a more normative alternative of operationalising responsiveness at the strategy-level, compared with a more procedural approach focusing on the R&D level. The healthy food criteria functioned as ‘downstream carriers’ of the normative goals to R&D operations. Like in Dutch food companies (Blok et al., 2015), the normative approach can be supported with some stakeholder engagement during early-phase R&D, and during later R&D phases with supervision by higher management that the stakeholder demands are taken into account.

With support of RRI literature, we can tentatively delineate benefits and risks of the more normative, strategy-level responsiveness. Regarding the benefits: clear strategic guidelines could help to sustain ethical aims, as the ethicality of the innovation lies less on the shoulders of individual teams and team members. Strategic guidelines can bring continuity, for example when an R&D project proliferates into several parallel trajectories (e.g. Datashare case: Noorman et al., 2017), or when the R&D team changes. Furthermore, a clear division of labour safeguards scarce resources: When societal goals are managed at the strategy level, R&D’s resources can be focused on techno-economic development. This may be particularly vital, when strict health criteria pose additional challenges for developing a techno-economically viable product (Blok et al., 2015). Furthermore, the public roles of higher managers can be more supportive to societal reflection. For example, CEO’s are expected to take public stands on wider issues regarding the companies’ activities (Asveld & Stemerding, 2017)

On the other hand, it is questionable to what extent the strategy-level alone can grasp societal impacts of innovations as future-oriented activity bound with uncertainties. In line with van de Poel (2017) and Asveld & Stemerding (2017): innovations uphold three types of uncertainties. Epistemological uncertainty arises from lack of knowledge, and can usually be reduced by further research at any phase. Indeterminate uncertainty is experienced when several options for the course of the innovation are still open, and can be resolved only as the innovation becomes ‘done’ and introduced in society. Ambiguous uncertainty arises from diverging viewpoints of societal actors on a specific topic, which are often of moral nature and thus hard to falsify or prioritise. In the FoP and Dutch food cases, the healthy food criteria appear to enjoy a broad societal consensus, making them societally representative guidelines. That is: the criteria appear objective (low epistemological uncertainty), applicable incrementally (low indeterminate uncertainty), and undisputed (low ambiguous uncertainty). From this viewpoint, there appears little uncertainty or ‘moral intensity’ (Blok et al., 2017; Jones, 1991) to incentivise companies to ethical reflections and stakeholder involvement during the R&D process.

##### Ambiguous uncertainty

However, in situations of high ambiguous uncertainty, a broader and more inclusive reflection on the guiding norms may become vital for the acceptability and overall success of the innovation. Disagreement about ‘right impacts’ of the innovation implies that existing normative guidelines may fall short of representing stakeholder perspectives and capturing societal concerns. This is a known risk when new and emerging technologies are applied in innovation (Owen et al., 2013; Swierstra & Rip, 2007). Novelties such as synthetic biology or nanotechnology can ‘rob moral routines’ and turn them into topics of deliberation and reconsideration (Swierstra & Rip, 2007). This was noted also by one of the Dutch food companies: when any emerging issue is involved that society is not widely familiar with (e.g. use of fish oil in foods), much more discussion is needed to develop health criteria that both companies and stakeholders can agree upon. In such cases, some companies also asked stakeholder opinions in the late (commercialisation) phase, as an ‘extra check’ that can have an impact on the market launch. (Blok et al., 2015)

Ambiguous uncertainty can also appear when innovations incorporate values that are prone to conflict, such as privacy and access (Noorman et al., 2017.; van den Hoven, 2013) or environmental qualities and economic competitiveness (Owen et al., 2012). Further, seemingly undisputed values may turn out to be ambiguous, such as ‘environmental friendliness’ in debates concerning shale gas exploitation (Dignum et al., 2016), or ‘sustainability’ in the Ecover case (Asveld & Stemerding, 2017). The latter describes two companies developing a bio-based detergent, which faced unexpected critique from a CSO, leading to the dismissal of the project near the product launch. While the companies assessed sustainability in terms of climate change mitigation, the CSO stressed impacts on biosafety (novel biotechnologies were involved), and socio-economic aspects of sustainability (negative impacts on third world farmers). The authors argue that stakeholder involvement would have revealed the differing understandings and value frames behind the seemingly uniform goal of sustainability, possibly saving the project. (Asveld & Stemerding, 2017).

##### Indeterminate uncertainty

Even when strategy-level decisions are furthered with stakeholder involvement in the early R&D phase, it may not suffice for addressing ambiguities. Indeterminate uncertainty implies that some ambiguities appear and become tangible only as the innovation proceeds (van de Poel, 2017). These ‘unknown unknowns’ are intrinsic to innovations (e.g. Pellizzoni, 2004), implying that we cannot fully know beforehand the extent of the unknown (Swierstra & Rip, 2007), and what all can go wrong (van de Poel, 2011). This indeterminacy appears the more pertinent, the more radical the innovation is: when the outcomes are not applicable with incremental changes to existing structures, practices and systems (Swierstra & Rip, 2007). Thus, whereas high ambiguity calls for societal deliberation on the ‘right impacts’ of an innovation, high indeterminacy suggests that such ambiguities may be best explored throughout the development process, as part of the hands-on R&D work.

##### Communicating uncertainties

Given that Corporate Social Responsibility (CSR) strategies often remain distant from R&D, further attention may be required to secure enough exchange of information between R&D and strategy management about ambiguous and indeterminate uncertainties. For example: do ‘organisational factors’ such as corporate structures and culture (Blok et al., 2017; Jones, 1991) also encourage communication ‘upstream’ – from R&D to strategy management? Active communication about successes and failures regarding normative guidelines along the R&D process could reduce the risk of the escalation of discrepancies between strategy and practice: for instance by exposing needs to readjust ‘downstream’ carriers like health criteria, or needs to reconsider the strategy.

*In summary:* With process-responsiveness, we suggest to consider the extent of uncertainty in weighing whether to open-up the innovation at (procedural) R&D level to joint societal reflections, and to communicate these uncertainties across the organisation. This could be considered as a step towards better dealing with unpredictable societal impacts of innovations, which CSR in its current form does not fully support (Pellé & Reber, 2015). However, since reduced uncertainty may not be the only benefit from opening-up, further discussion would be needed about the risk of overlooking other positive effects that deliberation on ethical and social issues can have on project management, personal motivation, or teamwork, among others (cf. Flipse, et al. 2013a).

### How to become mutually responsive: Product-responsiveness

#### Limitation: Fragile stakeholder relations

RRI expressly calls innovators and stakeholders to collectively reflect on the ‘right impacts’ and purposes of the innovation, and to jointly formulate its goals and directions. However, different understandings about the ‘right impacts’ can appear as *tensions* in stakeholder relations, limiting the innovators’ willingness to expose stakeholders to each other and to the innovation, in fear of risking the R&D project and outcome. Furthermore, the present case studies highlight that stakeholders are not always willing to get involved either. Stakeholders may be *indifferent,* indicating a difficulty to get them committed. At times, also the committed stakeholders may be *reluctant* to become too closely involved, in order to remain objective and neutral. Certain stakeholders may completely *avoid* collaboration with companies due to strategic reasons. Finally, *competitive* relations can emerge among actors with overlapping interests. If we are to open-up the innovation to deliberative participation, such fragilities in stakeholder relations challenge mutual responsiveness from several perspectives.

##### Tension

It is known in RRI that differing understandings (e.g. values and worldviews) can bring about ambiguous uncertainty, potentially manifesting as tensions between stakeholders (Asveld & Stemerding, 2017). Such tensions characterise the Datashare case from its inception. Datashare’s ‘privacy friendly data sharing platform’ was intended to simultaneously give control to residents over their own data, and to attract businesses interested in accessing personal data. As Noorman et al. (2017) note, values of privacy and autonomy ‘sit uneasy in the societal debate’ with those of accessibility, efficiency and profit. Direct contact with stakeholders was perceived as a substantial risk for the continuation of the project, making Datashare cautious to not bring together their business partners with the privacy-CSO’s. Datashare’s refusal to organise a stakeholder workshop contradicts with RRI’s strategies to ‘resolve tensions through explication of different perspectives and deliberation’ (Noorman et al., 2017). It appears questionable, whether seeking mutual responsiveness in form of e.g. aligned expectations, agreement on courses of action, or even agreeing to disagree, would have been possible without jeopardising the project.

Yet, value tensions were not the sole reason for Datashare to restrict stakeholder involvement. Similarly, while some of the Dutch food companies recognised ‘differing visions, goals, motives, sectors and values’ as critical issues, they brought out several other factor limiting interactions (Blok et al., 2015). While the attention within RRI has been steered towards value-laden tensions regarding ‘right impacts’ of innovations, the present case studies brought out a need to draw further attention also to the other fragilities in stakeholder activities, which in some situations can take priority.

##### Indifference

Commitment of stakeholders is an acknowledged requirement for successful collaborations (Blok et al., 2015; Flipse et al., 2014; Nielsen, 2016). Datashare innovators expressed that it was difficult to secure and maintain commitment of some of their business partners, who were not interested in privacy solutions and even less so in users’ control of data. As noted by Nielsen (2016), arguments for responsiveness often and misleadingly assume a mutual interest among the actors in the long-term robustness and desirability of the project. In contrast, for Datashare the relations with the indifferent (but strategically important) business partners appeared as ‘very fragile and in need of careful nurturing’. As a result, these stakeholders were not involved at early R&D steps, before there was something concrete to demonstrate to them (Noorman et al., 2017).

##### Reluctance

Further, stakeholders may be reluctant to get involved at certain steps of the innovation, for the sake of remaining neutral and independent. Dutch food companies rarely engaged stakeholders during the middle (product development) phase of R&D, and brought out that most stakeholders also wanted to step out before this phase, and instead take an external critical perspective. (Blok et al., 2015) This was one of the manifestations of a stark disparity between RRI’s ideas and practice in the case study: While it is assumed in RRI that mutual responsiveness leads to sharing responsibility, companies and their stakeholders appeared unanimous in their view that the company alone takes the responsibility for decisions, as the investor for risky, uncertain and costly innovation (Blok et al., 2015).

##### Avoidance

Moreover, critical stakeholders such as CSO’s may have strategic reasons to avoid any collaboration with the private sector, as this could endanger their credibility in the eyes of their sympathisers (Asveld & Stemerding, 2017; Blok & Lemmens, 2015). This may partly explain Datashare’s decision to not bring together their business partners and privacy activists. Datashare was also careful not to become too closely associated with either of them, in order to remain credible with both (Noorman et al., 2017).

##### Competition

While differences between stakeholders can cause tensions, much similarity can also complicate collaboration due to overlapping interests. Although there appeared no direct competition between the Dutch food companies and their non-commercial stakeholders, most companies were concerned that critical information could leak to their competitors through non-commercial stakeholders. As pointed out by Blok and Lemmens (2015) and Flipse et al. (2013b), concerns about the loss of competitive advantage in the private sector challenge the ideas of increasing transparency and reducing information asymmetries. Complementarily, one of the Dutch food companies expressed that differing interests (e.g. business profit and academic publications) can ease interactions among stakeholders (Blok et al., 2015).

### Suggestion: Product-responsiveness

While global challenges are collective concerns, the needs and interests of various stakeholder groups regarding these challenges can differ significantly. As we discussed over process-responsiveness*,* ambiguous uncertainty indicates a need for *opening-up* the innovation at the R&D-level to the deliberation on goals and purposes, which based on RRI’s ideas should involve both innovators and stakeholders. However, the very ambiguities complicate both the *opening-up* and *closing-down* of the innovation, so that during the R&D *throughput* (Blok & Lemmens, 2015), it can be difficult to reach a joint understanding about how to steer the innovation, and to formulate decisions that would be genuinely representative to stakeholder insights. Furthermore, apart from content-related tensions, various other fragilities in stakeholder relations contribute to a discontinuous and asymmetrical stakeholder participation.

Reflecting on the present case studies and previous RRI literature, we suggest to also consider the *output* of the innovation (Blok & Lemmens, 2015), such as a product or service, as one resource to operationalise responsiveness. **Product-responsive** innovation:

- Takes actions to open-up the innovation to stakeholder engagement during R&D, when *process-responsiveness* alerts of such need. Along with the option of closing-down during R&D:
- Considers the option of *open-ended products*, adaptable after the product launch according to diverging values, needs and interests. Approaches that may support in the design of such products include Value-Sensitive Design (VSD) and Adaptive Management.
- Is aware of the various fragilities in stakeholder relations, and considers the option to compensate asymmetries in stakeholder participation by increasing the possibility of choice (adaptability) in the final product.

We can hypothesise an example of product-responsiveness based on Datashare’s project: Privacy settings of the data sharing platform could be adjustable by resident-users, according to how comfortable they are with sharing their information. Acknowledging the option of open-ended products could temperate expectations for aligned stakeholder visions and joint understanding during the R&D process, perhaps encouraging to a more thorough opening-up. Further, product-responsiveness could perhaps compensate some of the asymmetry in stakeholder involvement, by allocating choice also to the less involved stakeholders. Product-responsiveness also makes RRI more explicit about possible roles for adopter-stakeholders, if they are to become mutually responsive with innovators ‘with a view to the societal aspects of the innovation’ (von Schomberg, 2013). Namely, the product may carry different stories and meanings to different users, who partake in the closing-down by adjusting the product. Thus, also the understanding of responsiveness as a *relation* between innovators and stakeholders becomes more diverse, giving space to more overlapping and ‘porous’ roles for producers and adopters.

#### Reflecting RRI and practice

Both the Dutch food companies and Datashare were actively involving stakeholders. The case studies capture two distinct approaches, and two problematics, in dealing with *tensions* stemming from deviating stakeholder needs and interests. First, Dutch food companies appear to be driven by the aim of *reducing ambiguity* through stakeholder engagement. They made attempts to align ‘expectations, experience and identity’ in working towards a joint vision about their innovations. On the other hand, Datashare appeared to *uphold ambiguity* during their stakeholder engagement*:* they were moulding several separate innovation trajectories, emphasising different aspects of their product to different stakeholders. To further explore these approaches, it appears useful to follow Stirling’s (2008) distinction between *appraisal* and *commitment* in the function of participatory deliberation.

##### Reducing ambiguity

While Dutch food companies placed importance on the formulation of shared objectives *(closing down commitment),* it remains an open question, to what extent the appraisal was opened-up for diverging discourses and framings at the beginning. What indicates *opening-up appraisal*: The companies had frequent meetings with several stakeholders, in formal and regular settings like project meetings, as well as more informal and irregular such as symposia. They emphasised among others the importance of sharing results, networking through multiple projects, and ad-hoc discussions about signals received from the market. They favoured directness and concreteness in stakeholder interaction, such as: ‘this is the product and this could be the package. What is your first impression?’. (Blok et al., 2015) However, the companies and their stakeholders appeared relatively unanimous already from the beginning. As discussed earlier, one foreseeable reason is the low uncertainty: Healthy food criteria are already widely accepted as guidelines for food innovations, and the health issues at stake (e.g. excessive use of salt) have already been broadly discussed in society (Blok et al., 2015). Yet, Blok and colleagues also reported a tendency to overcome uncertainties by the very selection of ‘aligned and complementary parties’, signalling *closing-down appraisal*. As one interviewee said, ‘I don’t really have experience with this [barriers related to different visions and missions among stakeholders] but if this is the case, we just search for another party with which we have a match’. In line with Blok and Lemmens (2015): closing-down appraisal can be a particular risk when the *input* of innovation process is in the global Grand Challenges, like public health. These challenges are ‘wicked problems’ (cf. Rittel & Webber, 1973), in that they are highly complex and not amenable for definite solutions. This makes agreeing on the problem definition highly challenging and prone to incumbent interests of powerful stakeholders, bringing the responsiveness towards stakeholders highly questionable (Blok & Lemmens, 2015). Further, regarding the food sector, Haen et al. (2015) and Swierstra & te Molder (2012) have remarked that certain concerns seem ‘structurally marginalized and barely recognized as legitimate public issues’ for deliberation, such as concerns related to naturalness, ownership and control, identity, and lifestyles.

##### Upholding ambiguity

Against this backdrop, Datashare innovators (Noorman et al., 2017) appear to have taken the challenge of *opening-up appraisal* of the innovation to differing and also conflicting stakeholder understandings. Their innovation invited tensions from the very outset of its idea (a platform integrating privacy and access), and the assembly of stakeholders, from whom they continuously gathered feedback for their prototype. However, Datashare responded to conflicting understandings by managing multiple innovation trajectories and maintaining their stakeholders separate, ‘without confronting them with the tension between the different perspectives on data sharing’ (Noorman et al., 2017). The innovators worked as translators between the stakeholders, by ‘carefully managing and cultivating the information’ obtained via different trajectories. For instance, for their business partners Datashare emphasised a more intimate contact with residents, whereas for privacy activists they highlighted how privacy can be integrated in the product design. On the one hand, this strategy enabled input from stakeholders, who perhaps would have refused to directly collaborate with each other, due to for example strategic reasons. Yet, it remains an open question how the trajectories would be closed-down at the *throughput* of the R&D (*closing-down commitment*), so that the platform would eventually accommodate the conflicting needs and interests. Can Datashare remain responsive to both their business partners and privacy-activists?

##### Other fragilities in stakeholder relations

In addition to these content-related tensions, both Dutch food companies and Datashare were experiencing other fragilities in stakeholder relations, which further complicated both *input* and *throughput*. As a result, stakeholders were not equally involved and informed in every phase. As per Blok & Lemmens (2015), such information asymmetries during R&D make mutual responsiveness questionable. However, Datashare and the Dutch food companies had management practices for enabling stakeholder collaboration *despite* of various fragilities – even if the outcome was not ideally ‘symmetrical’. For instance, when companies faced difficulties in raising some of their stakeholders’ interest, they were nevertheless able to involve the more devoted ones (i.e. managing with *indifference*). Further, companies made efforts to further interest their stakeholders with ‘socialisation mechanisms’, including formal regular project meetings and more informal events like symposia (*indifference*). When stakeholders wished to stay neutral during the middle phase of R&D, companies and stakeholders jointly agreed that the latter step out after the early R&D phase (*reluctance*). Bilateral meetings with strategically divided stakeholders (instead of multi-stakeholder collaboration) enabled their input in the first place (*avoidance*). Companies applied protection mechanisms to secure crucial information, including formal mechanisms like intellectual property management, and semi-formal such as confidentiality agreements (*competition*). As any formal mechanism has its limitations, they also highlighted the importance of building mutual trust and open organisational culture (*competition*). (Blok et al., 2015; Noorman et al., 2017) Nevertheless, some asymmetry remained despite management practices, further questioning to what extent the appraisals and commitments were representative to societal needs.

To recap: While our suggestion for process-responsiveness stemmed from the challenge that opening-up does not often occur at the R&D level, three further challenges regarding mutual responsiveness appear where such opening-up is (allegedly) ensued. First, innovation is only selectively opened-up for the *input* (indicating *closing-down appraisal*). Second: when *opening-up appraisal* results in conflicting advice, how to reach a *closing-down commitment* during *throughput*? Third, in addition to content-related tensions, coping with other fragilities leaves residual asymmetries, further questioning whether the innovation is representative of societal needs.

##### Open-ended products

As mutual responsiveness regarding the *input* and *throughput* of the innovation has been already problematized (Blok & Lemmens, 2015), we suggest giving consideration also to the *output* of innovation as a resource for responding to diverging societal needs. That is: to extend the scope of responsiveness into opportunities that innovations uphold once they are ‘out in the world’ (Robaey & Simons, 2015) after the market launch. Out of their developers’ immediate presence, these outputs are not only applied by some of the stakeholders, but possibly also modified further to better fit the context of their use. These post-launch developments can be left overlooked, when innovation is conceptualised as a process starting from the ideation and ending to the launch (e.g. stage-gate model). Does such a conceptualisation also contribute to ‘undue privileging’ of *closing-down* commitment (Stirling, 2008) in RRI, possibly discouraging from genuinely opening-up the innovation to differences? What opportunities there appear for *open-ended commitments* (Stirling, 2008), acknowledging and even inviting stakeholder responses via post-launch modifications?

It is not far-fetched to envision that Datashare’s platform could eventually allow each resident-user to adjust their own privacy settings, according to how comfortable they are with sharing energy consumption data. Also, RRI theory and associated approaches seem to encourage further contemplations on the potential of open-ended products in enhancing responsiveness. For instance, RRI’s definition by von Schomberg (2013) calls societal actors and innovators to ‘become mutually responsive to each other with a view to the … innovation process *and its marketable products*’ (emphasis added), while van den Hoven (2012) discusses the potential of technologies to spawn new moral choice situations. Concerning different approaches, *value-sensitive design* (Friedman, 1996) has been proposed in RRI for the design of products (e.g. van den Hoven, 2013), and processes (e.g. Dignum et al., 2016), and as such it is a means to operationalise moral choice. Furthermore, *adaptive management* (Armitage et al., 2008) has been linked to RRI as a means to resolve conflicting stakeholders claims, by developing innovation outputs that incorporate multiple trajectories that are switchable or adjustable after launch if unwanted effects appear. An example of this is provided by Asveld and Stemerding (2017), hypothesising an adaptable bio-process as an alternative ending for the Ecover case, able to switch between various feedstocks in case the sustainability of a particular feedstock is later confronted. This could provide a ‘way out’ from a particular trajectory (e.g. use of particular feedstock), thus avoiding stranding the innovation into a deadlock. Another variation of adaptive design could be the hypothesized output from the Datashare platform, in which different options are left open so that they are applicable in parallel, without excluding some or any of the options.

Finally, incorporating a spectrum of options in the final product could increase resilience in face of asymmetrical stakeholder participation. Although open-ended outputs may not fully compensate the information and power asymmetries, they could at least allocate some more choice also to the less involved stakeholders. In this sense, open-ended outputs may increase ‘porosity’ of innovation structures (Pavie et al., 2014) against power asymmetries – while broadening discourses from ‘who dominates whom’ (Pavie et al., 2014) and from ‘cultural expectations for proponents and opponents’ (Swierstra & Rip, 2007), also towards more many-sided and proactive roles for producers and adopters. *In summary:* With product-responsiveness, we suggest to consider also open-ended commitments, in addition to closed-down commitments, as a resource for operationalising responsiveness. Yet, along with the opportunity of increasing users’ choice, further discussion should also follow about the trade-off of increasing complexity. Blok and Lemmens (2015) remind that innovation *outputs* uphold radical uncertainty, as our knowledge about the impacts of innovations is limited in general, and especially so when the input is in the Grand Challenges that have no straightforward solutions. Further, van den Hoven et al. (2012) elaborate that when (moral) choice is increased with new technology, we become faced with new side effects and risks, stirring up new value dimensions and again more choice situations (to be tackled with e.g. further technology development). This considered: How then does increasing choice in the output affect the acceptability, sustainability, or distribution of accountability – and the ‘freedom of choice’ per se – when individual choices are considered in terms of their collective impacts, or when immediate benefits turn into long-term impacts? Such questions are becoming increasingly tangible, as in sectors like ICT the ‘smart and flexible’ (customisable) products and services already outnumber single-interface alternatives (Keates, 2015). RRI can foster discussion on both ‘right impacts’ and risks of such products.

### With whom to become mutually responsive? *Pre*sponsiveness

#### Limitation: No perceived help from society

During early R&D, there were occasions when innovators perceived a need for societal insight, but experienced that stakeholder engagement would not provide tangible contributions for steering the innovation. *No input* either from stakeholders or the innovators themselves was considered meaningful for a fruitful interaction. Datashare innovators expressed that they had not much to get from potential resident-users, regarding privacy concerns and expectations related to their product idea. Simultaneously, the innovators had not much to give either, as the vision for the data sharing platform was not yet clear. (Noorman et al., 2017) The innovators believed that end users have ‘latent needs’ for privacy, which are difficult to discuss without providing them a clear idea. As one Datashare team member reflected: When people are asked whether they are concerned about their data, they will say no, but in the context of a concrete example they may give a different answer. Further, the team members felt there were *not enough resources* (time) to explain their concept to the resident-users in its current undeveloped state, as Datashare’s funders expected the team to proceed quickly (Noorman et al., 2017). Moreover, as Datashare was still reviewing several options for further development of their innovation, Noorman et al. (2017) remark that it may have also been *difficult to identify* relevant stakeholders in the first place.

#### Suggestion: *Pre*sponsiveness

As we discussed over process-responsiveness, indeterminate uncertainties call for stakeholder involvement all along the R&D process, as the ‘points of interruption and control’ of such uncertainties are highly diffuse over time and space (Lee & Petts, 2013). In private sector, indeterminate uncertainty seems to entail a two-fold challenge: While it is generally problematic to grasp the impacts of an innovation during early-phase R&D (the Collingridge dilemma), innovators are nevertheless expected to quickly yield tangible results.

From the perspective of mutual responsiveness, the Collingridge dilemma signals an indeterminate uncertainty that all relevant stakeholders may not be known at the time of R&D. Yet, responsiveness as a future-oriented responsibility obliges a receptive attitude towards the needs and desires of others, before deciding what to do (Pellizzoni, 2004). If we are to open-up the innovation to participatory deliberation, who exactly should be involved? Furthermore, how to be responsive to those actors that are potentially affected by the innovation, but are not available at the context of R&D? We suggest a ***pre*** **sponsive** approach, which:

- Is aware that relevant stakeholders can be unknown and unreachable at a given time of R&D. Among others, stakeholders can be distant in time, place, or sector.
- Takes actions to identify unknown stakeholders and their needs. For example, as part of the experimental approach to innovation.
- Critically reflects on the representativeness of mediators (e.g. interest groups and experts) to stakeholder needs and interests.

*Pre*sponsiveness further elaborates responsiveness as *forward-looking responsibility:* While the first step is to acknowledge that there are uncertainties regarding stakeholders, the receptive attitude should also result in efforts to identify stakeholders and their needs, so that mutual relations could be (at some point) established. However, there is little practical advice derivable from the case studies on how to achieve this. Nevertheless, we have identified *experimentation* as a promising approach in the private sector to address stakeholder-related uncertainties along with other (indeterminate) uncertainty.

#### Reflecting RRI and practice

Datashare’s experiences during early R&D echo with the Collingridge dilemma (Collingride 1980). At the time when the concept for Datashare’s platform would still be amenable to modifications based on the input from resident-users, there is not enough knowledge for grasping the societal impacts of the innovation. Yet, by the time the concept would be explicit enough to allow diverse societal reflections, it is already locked-in to certain trajectories so that steering the innovation is difficult, costly and time consuming (e.g. Flipse et al., 2013b; Noorman et al., 2017; Owen et al., 2012) Moreover, the dilemma seemed to be exacerbated by the constant pressure from funders to rapidly produce a proof of demand for the product, driving Datashare to proceed while the long-term picture was not yet clear (Noorman et al., 2017). In the private sector, tight schedules commonly challenge appropriate monitoring of uncertainties (Pavie et al., 2014). Stakeholder interactions are time-consuming, and within a short time it is difficult to have a fruitful exchange of thoughts about the purposes of the innovation (Blok et al., 2015; Lee & Petts, 2013; Noorman et al., 2017). Especially in start-ups, like Datashare, resources are scarce and tightly steered at securing market entry. Hence, start-ups need to carefully balance the claimed benefits of stakeholder engagement with costs and launch delay. Still, start-ups often work with new and emerging technologies, which specifically calls for timely stakeholder discussions. (Scholten & van der Duin, 2015)

##### Experimentation

Facing pressures for a quick proof of demand, Datashare innovators found themselves looking for ‘evidence for something that did not exist yet’ (Noorman et al., 2017). In order to work toward this evidence, the team got inspiration from the Lean (start-up) method (cf. Ries, 2011). In a Lean R&D, a prototype or a proposition is modified iteratively, in short cycles of ‘validated learning’. Feedback from customers is frequently gathered and applied to further refine the prototype. (Noorman et al. 2017) With this focus, the Lean method resembles the *experimental approach* to innovation, described as continuous testing and learning by means of gradual scaling-up, *while* a technology is introduced in society (e.g. Asveld, 2016; Robaey & Simons, 2015; van de Poel, 2011). Experimentation can be perceived as an effort to manage with the trade-offs resulting from the Collingridge dilemma. First, it is acknowledged that due to uncertainties, meticulous plans are unfeasible in the early steps. Second, the focus is on the discovery and management of uncertainties as they appear along the project: before the innovation is introduced to society in its full scale with possible broad negative impacts. (Asveld, 2016; Van de Poel, 2017).

It has been suggested that experimental approach can support integration of various RRI principles into R&D processes (e.g. Asveld, 2016; Robaey & Simons, 2015.; van de Poel, 2011) – also in the private sector as experimentation yields gradual results along the R&D process, satisfying investors’ expectations for a quick evidence (Noorman et al., 2017). Among others, experimentation involves frequent collaboration with societal actors, supporting mutually responsive relations. More specifically: experimentation explicitly includes the aim of learning (i.e. not only gathering information from stakeholders), it supports exploration of different interpretations on the innovation (opening-up appraisal), and on how values might evolve owing to its introduction (society’s responses). Further, stakeholders can be given a chance to step out of the experiment, and to influence on the set up, carrying out, and stopping the experiment (impact on innovation trajectory). However, as van de Poel (2017) also points out, following an experimental method in R&D does not self-evidently lead to a *responsible* conduct of experimentation. From the perspective of mutual responsiveness, in the case studies we can distinguish a challenge regarding unreachable stakeholders, most explicitly in relation to ‘mediated presence’ (representativeness).

##### Unreachable stakeholders

Noorman et al. (2017) indicate that further involvement of stakeholder groups in the Lean method may have been limited by a difficulty to identify or specify relevant stakeholders. While it was not explicit to what extent Datashare’s innovators were aware or concerned about this limitation, RRI literature identifies multiple reasons for why stakeholders can be ‘unreachable’. Based on the background case studies, we distinguished four such circumstances. First, potential stakeholders can be *distant in time* of the R&D: either not yet identified as stakeholders, or belonging to future generations (e.g. Balkema & Pols, 2015). This challenge of responding to future stakeholders is essentially linked to the definition of sustainability (Brundtland, 1987) and intergenerational justice (e.g. Pols & Spahn, 2015). Second, stakeholders may be geographically *distant in place*, and yet being increasingly interconnected via complex supply chains (e.g. Balkema & Pols, 2015), or digital technologies (e.g. Nevejan & Brazier, 2015). Third, and often related to geographical distance, stakeholders with very different backgrounds can be *distant in discourse,* e.g. due to sectoral differences (Blok et al., 2015), different cultural and national settings (Lee & Petts, 2013), or levels of education (Asveld & Stemerding, 2017). For instance, small-farmers in developing countries might be among the most challenging stakeholders to involve in stakeholder interaction (Asveld & Stemerding, 2017; Balkema & Pols, 2015).

##### Mediated presence

Fourth, in all of the above examples, absent stakeholders can be represented by mediators such as interest groups or experts (e.g. Delgado et al., 2011; Stirling, 2008). For example, Asveld & Stemerding (2017) note that CSOs readily take the role of speaking on behalf of small-farmers, who themselves remain largely unheard. Also, how Datashare team approached the evasive ‘latent privacy needs’ of resident-users through the Lean method led Noorman and colleagues to contemplate on the ‘objectified’ role of this stakeholder group. User preferences were made explicit via ‘multiple translation steps’, so that the team first consulted external experts, who examined citizens’ perceptions about privacy – either directly (interviews) or indirectly (media analyses). In addition, the Datashare team reflected on their own stances to privacy as ‘average potential users’. Partly based on these inputs, the team then developed prototypes that were ‘validated and refined’ with focus groups recruited by an agency. In the meanwhile, Datashare involved particularly interested stakeholder groups more directly, thus giving more weight to some of potential business partners and to an extent to privacy CSOs. Consequently, the resident-users had less impact on the problem-setting: In focus-groups, they were given roles as representatives of certain perspectives on the prototype that already incorporated a limited number of options. (Noorman et al., 2017)

As regards stakeholder representation, Stirling (2008) has noted that indirect expert analysis is not self-evidently less ‘conductive to enhanced social agency’ than participatory deliberation in every circumstance. Also, it is known to be challenging to arrange a reasonably manageable but not too homogenous amount of design options in practice (Keates, 2015). Nevertheless, the case studies indicate a need to be at least aware that relevant stakeholders may be absent and unknown during R&D. This further attention is justified not least by the tendency to define technological opportunities more clearly for certain stakeholders, while harms remain speculative and farther away, concerning ‘as yet anonymous, collective stakeholders’ (Swierstra & Rip, 2007). To employ such awareness for enhancing stakeholder representativeness: Asveld and Stemerding (2017) suggest that experimenting with *worldviews* (cf. Hedlund-de Witt, 2013) could have been applied in the Ecover case during early R&D, in order to grasp different perspectives on ‘sustainability’ already before direct stakeholder involvement. The identified perspectives and tensions regarding a specific topic can be connected to a manageable number of *worldviews*: a systematically assembled set of coherent value structures shared by a wide range of people in society. If the identified perspectives cover all these worldviews, it can be an indication that representation is sufficient (Asveld & Stemerding, 2017; Hedlund-de Wit, 2013). A similar experiment could be hypothesised for Datashare regarding stakeholder perspectives on ‘privacy’, e.g. as a pre-step for further focus-group work.

*In summary*: With *pre*sponsiveness, we draw further attention to stakeholders, who despite their current absence may still be affected by, or contributing to, the innovation at its later steps. With the exception of the worldview approach, there is little practical advice in the present case studies for how to identify the needs or identities of these stakeholders. However, experimental approach appeared as a potential ground in the private sector for further addressing stakeholder-related uncertainties, along with other (indeterminate) uncertainty.

## Conclusions

This paper is an early attempt to further elaborate RRI’s concept of responsiveness based on recent practical examinations in private sector R&D. We took a mind-set that tensions between theoretical ideals and complex realities are creative tensions, ‘inspiring innovation, experimentation, and future research into alternative options and solutions’ (Delgado et al., 2011). Inspired by limitations of mutual responsiveness, we first propose *process-responsiveness:* an elaboration of responsiveness as the *action-element* of RRI that triggers attention to societal uncertainties, which particularly call for R&D-level opening-up. With this proposition, we hope to contribute to the further research on interactions between CSR and R&D, while acknowledging a need for more discussion: reducing uncertainty is hardly the only possible benefit following from opening-up. With *product-responsiveness,* we encourage to consider the option of ‘open-ended products’ in operationalising responsiveness to diverse societal needs. While product-responsiveness can diversify the understanding of responsiveness as a *relation* between producers and adopters, we also acknowledge needs for further discussions regarding the trade-off of increasing complexity. Finally, we suggest pre*sponsiveness* as an expression of responsiveness as *forward-looking responsibility*, drawing attention to stakeholders whose unavailability at a given moment does not per se make them any less significant. While *pre*sponsiveness largely remains an open challenge, we identify experimentation as one starting point for identifying unavailable stakeholders and their needs.

We cautiously remark that these suggestions are not intended for downplaying the importance of ‘ideal-type’ mutual responsiveness for responsible innovations, for undermining more refined conceptualisations of mutual responsiveness, or for giving reasons to neglect stakeholder involvement. It is rather our purpose to envision complementary – and perhaps alternative – modes to be responsive to societal needs, which are also not too far-fetched regarding RRI’s own theories. Finally, we realise that due to the limited number of available case studies, further research is needed. Our analysis incorporates different cases and contexts, without closely considering the significance of their difference to the identified opportunities and limitations. With this remark, we refer to the diversity in sectors (food, ICT), types of companies (mature, start-up), set-ups for R&D activities (e.g. tasks of researchers), and stakeholders (research organisations, CSOs, business partners, consumers). More studies will make a more context-specific analysis possible.

## Abbreviations

CSO: Civil society organisation
CSR: Corporate Social Responsibility
ELSA: Ethical, Legal and Social Aspects
R&D: Research and development
RRI: Responsible research and innovation
VSD: Value sensitive design.

## References

[CR1] Armitage D, Marschke M, Plummer R (2008). Adaptive co-management and the paradox of learning. Glob Environ Chang.

[CR2] Asveld L (2016). The need for governance by experimentation: The case of biofuels. Sci Eng Ethics.

[CR3] Asveld, L., Stemerding, D. (2017). Social learning in the bioeconomy: The case of Ecover. In I. Van de Poel, L. Asveld, D. Mehos. Experimentation beyond the laboratory: New perspectives on technology in society. London: Ashgate Publishers. (In press).

[CR4] Balkema, A., Pols, A. (2015). Biofuels: Sustainable innovation or gold rush? Identifying responsibilities for biofuel innovations. In B. J. Koops, I. Oosterlaken, H. Romijn, T. Swierstra, J. van den Hoven. Responsible innovation 2: Concepts, approaches, and applications (pp. 283–303). Dordrecht: Springer International Publishing. http://doi.org/10.1007/978-3-319-17308-5

[CR5] Blok V (2014). Look who’s talking: Responsible innovation, the paradox of dialogue and the voice of the other in communication and negotiation processes. Journal of Responsible Innovation.

[CR6] Blok, V., Lemmens, P. (2015). The emerging concept of responsible innovation. Three reasons why it is questionable and calls for a radical transformation of the concept of innovation. *Responsible Innovation 2: Concepts, Approaches, and Applications*, 19–35. http://doi.org/10.1007/978-3-319-17308-5

[CR7] Blok V, Hoffmans L, Wubben EFM (2015). Stakeholder engagement for responsible innovation in the private sector: Critical issues and management practices. Journal on Chain and Network Science.

[CR8] Blok, V., Tempels, T., Pietersma, E., Jansen, L. (2017). Exploring ethical decision making in responsible innovation: The case of innovations for healthy food. In L. Asveld, M. E. C. Van dam-Mieras, T. Swierstra, S. A. C. M. Lavrijssen, C. A. Linse, & J. Van de hoven (Eds.), *Responsible Innovation 3: A European Agenda?* Springer. (In press)

[CR9] Brundtland GH (1987). Our common future. World commission on environment and development.

[CR10] Cooper R (1990). Stage-gate systems: A new tool for managing new products. Business Horizons.

[CR11] Delgado A, Kjølberg KL, Wickson F (2011). Public engagement coming of age: From theory to practice in STS encounters with nanotechnology. Public Underst Sci.

[CR12] Dignum M, Correlje A, Cuppen E, Pesch U, Taebi B (2016). Contested technologies and Design for Values: The case of shale gas. Sci Eng Ethics.

[CR13] Flipse SM, van der Sanden MCA, Osseweijer P (2013). Midstream modulation in biotechnology industry: Redefining what is “part of the job” of researchers in industry. Sci Eng Ethics.

[CR14] Flipse SM, van der Sanden MCA, Osseweijer P (2013). The why and how of enabling the integration of social and ethical aspects in Research and Development. Sci Eng Ethics.

[CR15] Flipse SM, Sanden MCAVD, Radstake M, Winde JHD, Osseweijer P (2014). The DNA of socially responsible innovation. EMBO Rep.

[CR16] Flipse SM, Van Dam KH, Stragier J, Oude Vrielink TJC, Van der Sanden MCA (2015). Operationalizing responsible research & innovation in industry through decision support in innovation practice. Journal on Chain and Network Science.

[CR17] Friedman B (1996). Value-Sensitive Design. Interactions.

[CR18] Ganzevles, J, R van Est. 2012. PACITA: Collaborative project on mobilisation and mutual learning actions in European Parliamenary technology assessment – Deliverable 2.2. TA Practices in Europe Rathenau Instituut, 238. Hague: Rathenau Instituut.

[CR19] Haen, D., Sneijder, P., te Molder, H., Swierstra, T. (2015). Natural food: Organising “responsiveness” in responsible innovation of food technology. In B. J. Koops, I. Oosterlaken, H. Romijn, T. Swierstra, J. van den Hoven Responsible innovation 2: Concepts, approaches, and applications (pp. 161–181). Dordrecht: Springer International Publishing. http://doi.org/10.1007/978-3-319-17308-5

[CR20] Hedlund-de Witt, A. (2013). Worldviews and the transformation to sustainable societies: An exploration of the cultural and psychological dimensions of our global environmental challenges. Ph.D. Thesis. Amsterdam: VU University.

[CR21] Iatridis, K, D Schroeder.2016 Research and innovation in industry: The case for corporate responsibility tools: Springer http://doi.org/10.1007/978-3-319-21693-5.

[CR22] Jones TM (1991). Ethical decision making by individuals in organizations : An issue-contingent model. Acad Manag Rev.

[CR23] Keates, S. (2015). Design for the Value of inclusiveness. In J. van den Hoven, P. E. Vermaas, I. Van de Poel Handbook of ethics, values, and technological design (pp. 383–402). Dordrecht: Springer. http://doi.org/10.1007/978-94-007-6970-0

[CR24] Koops, B. J. (2015). The concepts, approaches, and applications of responsible innovations: An introduction. In B. J. Koops, I. Oosterlaken, H. Romijn, T. Swierstra, J. van den Hoven Responsible innovation 2: Concepts, approaches, and applications (pp. 1–15). Dordrecht: Springer International Publishing. http://doi.org/10.1007/978-3-319-17308-5

[CR25] Landeweerd, L. 2017. Europe by nature: RRI in a blurred European governance stucture Retrieved February 7, 2016, from https://www.researchgate.net/publication/313419598_Europe_by_Nature.

[CR26] Lee, R. G., & Petts, J. (2013). Adaptive governance for responsible innovation. *Responsible Innovation: Managing the Responsible Emergence of Science and Innovation in Society*, (august), 143–164. http://doi.org/10.1002/9781118551424.ch8

[CR27] Nevejan, C., Brazier, F. (2015). Design for the Value of presence. In J. van den Hoven, P. E. Vermaas, I. Van de Poel Handbook of ethics, values, and technological design: Sources, theory, values and application domains (pp. 403–430). Dordrecht: Springer. http://doi.org/10.1007/978-94-007-6970-0

[CR28] Nielsen, M. V. (2016). The concept of responsiveness in the governance of research and innovation. *Science and Public Policy*, scv078. http://doi.org/10.1093/scipol/scv078

[CR29] Noorman, M, T Swierstra, D Zandbergen.2017 *Responsible Innovation 3: A European Agenda?* In Reassessing the normative core of RI: The challenges posed to stakeholder engagement in corporate setting, ed. L Asveld, MEC Van dam-Mieras, T Swierstra, SACM Lavrijssen, CA Linse, J Van de hoven, editors: Springer (In press).

[CR30] Owen R, Macnaghten P, Stilgoe J (2012). Responsible research and innovation: From science in society to science for society, with society. Sci Public Policy.

[CR31] Owen, R., Stilgoe, J., Macnaghten, P., Gorman, M., Fisher, E., Guston, D. (2013). A framework for responsible innovation. *Responsible Innovation: Managing the Responsible Emergence of Science and Innovation in Society*, (august), 27–50. http://doi.org/10.1002/9781118551424.ch2

[CR32] Pavie, X, V Scholten, D Carthy.2014 Responsible innovation: From concept to practice: World Scientific Publishing Company.

[CR33] Pellé S, Reber B (2015). Responsible innovation in the light of moral responsibility. Journal on Chain and Network Science.

[CR34] Pellizzoni L (2004). Responsibility and environmental governance. Environmental Politics.

[CR35] Pols, A., & Spahn, A. (2015). Design for the Values of Democracy and Justice. In Handbook of Ethics,Values, and Technological Design: Sources, Theory, Values and Application Domains (pp. 335–363). http://doi.org/10.1007/978-94-007-6970-0

[CR36] Ries E (2011). How today’s entrepreneurs use continuous innovation to create radically successful businesses. The lean startup.

[CR37] Rittel HWJ, Webber MM (1973). Dilemmas in a general theory of planning. Policy Sci.

[CR38] Robaey, Z., Simons, A. (2015). Responsible management of social experiments: Challenges for policymaking. In B. J. Koops, I. Oosterlaken, H. Romijn, T. Swierstra, J. van den Hoven Responsible innovation 2: Concepts, approaches, and applications (pp. 87–104). Dordrecht: Springer International Publishing.

[CR39] Scholten VE, van der Duin PA (2015). Responsible innovation among academic spin-offs: How responsible practices help developing absorptive capacity. Journal on Chain and Network Science.

[CR40] Stilgoe, J., Owen, R., Macnaghten, P. (2013). Developing a framework for responsible innovation. Res Policy, 42, 1568–1580. http://dx.doi.org/10.1016/j.respol.2013.05.008

[CR41] Stirling A (2008). "opening up" and "closing down": Power, participation, and pluralism in the social appraisal of technology. Sci Technol Hum Values.

[CR42] Swierstra T, Rip A (2007). Nano-ethics as NEST-ethics: Patterns of moral argumentation about new and emerging science and technology. NanoEthics.

[CR43] Swierstra, T, H te Molder.2012 Risk and soft impacts, Epistemology, Decision Theory, Ethics, and Social Implications of Risk. In Handbook of Risk THeory, ed. S Roeser, R Hillerbrand, P Sandin, M Peterson, editors, 1049–1066: Springer http://doi.org/10.1007/978-94-007-1433-5.

[CR44] van de Poel I (2011). Nuclear energy as a social experiment. Ethics, Policy & Environment.

[CR45] van de Poel, I. (2017). Society as a laboratory to experiment with new technologies. In D. Bowman,E. Stokes, A. Rip (Eds.), Embedding new technologies into society: A regulatory, ethical and societal perspective. (pp. 61–68). Singapore: Pan Stanford Publishing.

[CR46] van den Hoven, J, GJ Lokhorst, I Van de Poel. 2012. Engineering and the problem of moral overload. Sci Eng Ethics 18(1): 143–155 http://doi.org/10.1007/s11948-011-9277-z. 10.1007/s11948-011-9277-z PMC3275721 21533834

[CR47] van den Hoven, J. 2013. Value sensitive design and responsible innovation. In Responsible Innovation: Managing the Responsible Emergence of Science and Innovation in Society, ed. R Owen, J Bessant, M Heintz, editors, 75–84. London: Wiley.

[CR48] Von Schomberg R (2013). Responsible Innovation*:* Managing the Responsible Emergence of Science and Innovation in Society. A vision of responsible research and innovation.

[CR49] Zwart H, Landeweerd L, van Rooij A (2014). Adapt or perish? Assessing the recent shift in the European research funding arena from “ELSA” to “RRI.”. Life Sciences Society and Policy.

